# Both Zn biofortification and nutrient distribution pattern in cherry tomato plants are influenced by the application of ZnO nanofertilizer

**DOI:** 10.1016/j.heliyon.2022.e09130

**Published:** 2022-03-22

**Authors:** Patricia Almendros, Demetrio González, María Dolores Fernández, Concepción García-Gomez, Ana Obrador

**Affiliations:** a Universidad Politécnica de Madrid (UPM), Chemical and Food Technology Department, CEIGRAM, Research Centre for the Management of Agricultural and Environmental Risks, Madrid, 28040, Spain; b Centro Nacional Instituto de Investigación y Tecnología Agraria y Alimentaria (INIA-CSIC), Environment and Agronomy Department, Ctra. A Coruña, km 7.5, 28040, Madrid, Spain

**Keywords:** Nanoparticles, ZnO, Tomato fruit yield, Nutrient concentrations, Micronutrient concentrations, Nutrient translocation

## Abstract

A pot experiment was conducted to determine the influence of commercial nanoparticles (ZnO-NPs) at different doses for use as nanofertilizer on nutrient uptake and its distribution in cherry tomato (*Solanum lycopersicum* L var. cerasiforme) plants in an acidic (soil pH 5.5) and calcareous soil (soil pH 8.5) from the Mediterranean area. We determined crop yield; macro- (N, P, K, Mg, S and Ca) and micro-nutrient (B, Cu, Fe, Mn, Na and Zn) concentrations in the different parts of the crop (root, stem, leaves and tomato fruits) and the extent of nutrient translocation to the aerial part of the plant. The concentrations of macronutrients N, P, K and Mg in tomato fruits grown in both soils can be considered adequate in terms of nutritional requirements. However, the Ca concentration in tomato fruits grown in the calcareous soil did not reach the required concentration to be considered sufficient. This effect was related to the characteristics of this calcareous soil. Although different concentrations of ZnO-NPs did not affect Fe and Na concentrations in tomato fruit, B concentration in tomato fruits increased with the application of ZnO-NPs. In addition, Cu concentration decreased with the application of ZnO-NPs compared to treatments without any Zn application (Nil-ZnO NP) in the calcareous soil. Manganese concentrations decreased with ZnO-NPs application in both soils. The effect of the application of ZnO-NPs depends on soil characteristics. Zinc applied as a nanofertilizer in the form of ZnO-NPs can be used to increase the crop yield and to obtain an adequate Zn biofortification in cherry tomato crop. The Zn concentrations in tomato fruits reached ranges of 4.5–4.8 mg Zn kg^−1^ in the acidic soil and 2.5–3,5 mg Zn kg^−1^ in the calcareous soil. Nutrient concentrations in these fruits following biofortification are adequate for human consumption.

## 1 Introduction

Zinc deficiency in humans is associated with diet quality and is aggravated by Zn-deficient soils (Alloway 2013). Zinc is an essential trace element and one which is of fundamental importance for the functions of over 300 enzymes and hormones of human body. Studies carried out by the Food and Agriculture Organization (FAO) have shown that Zn deficiency is the most common micronutrient deficiency and that it affects a wide range of soil types and is found in many different agricultural areas (FAO/WHO 2005; FAO 2017).

Using agronomic strategies to increase mineral element concentrations in the edible parts of crops is commonly known as agronomic biofortification. The biofortification of Zn has been highlighted as a promising way to accumulate high concentrations of Zn in grains or fruits and thereby alleviate human health problems associated with insufficient intakes of this micronutrient (Noulas et al., 2018).

Zinc is essential for plant nutrition and is regarded as a fundamental component of several enzyme systems. This micronutrient is the only metal that is required in all six enzyme classes and contributes to growth regulation, protein synthesis, energy production, enzyme activation, gene expression, phytohormone activity photosynthesis, carbohydrate metabolism, fertility, seed production and defense against disease (Hafeez et al., 2013; Sandeep et al., 2019). The metabolism of proteins, carbohydrates and auxin, and the correct functioning of reproductive processes are all adversely affected by a lack of Zn (Sadeghzadeh 2013). Zinc deficiency may cause physiological stress in plants given that Zn plays a fundamental role in many metabolic processes. Significant decreases in growth and fruit yield under Zn-deficient conditions have been widely reported (Karimi et al., 2019; Obrador et al., 2021).

Several Zn sources have been used to correct Zn deficiencies or biofortify different crops. These Zn sources include Zn sulphate, Zn oxide, Zn chloride, Zn nitrate, Zn oxy-sulphate or complexes and chelates of natural or synthetic origin. An appropriate correction results in an optimum yield and enhance fruit plant quality (including a suitable concentration of nutrients) (Pal et al., 2021). The benefits obtained from adequate fertilization will depend on the nature of the fertilizer, the soil characteristics, the cultivar and the crop system employed (Gonzalez et al., 2019a). The physicochemical properties of different Zn fertilizers, including solubility, chemical composition, shape, agglomeration state, crystal structure or surface energy, affect processes such as the adsorption, aggregation, dispersion and solubility of particles in soil (Tiede et al., 2008). Therefore, these fertilizer properties influence the nutritional quality and yield potential of the crops grown.

In recent years, the possible use of nanoparticles (NPs) as nanofertilizers has been considered as an alternative to the use of traditional fertilizer (Rasli et al., 2020; Shebl et al., 2020). Nanoparticles of metal oxides are gradually being incorporated into agricultural products, including fertilizers, although only at an experimental phase. The NPs possess unique physical and chemical properties due to their high surface area and nanoscale size. Singh (2018) found that although the water solubility of Zn oxides is lower than that of other Zn sources such as Zn sulfate, the ZnO-NPs show an amount and speed of dissolution considerably higher than bulk fertilizers due to the reduced particle size and greater specific surface area.

As is well known, adequate fertilization and a balanced supply of nutrients are important factors to achieve an optimum crop yield and quality (Haider et al., 2020). Interactions between macronutrients and/or micronutrients affect their concentrations in plants (ionome) and the quality of the fruit, with potential impacts on human nutrition. Such interactions may take place in the soil and within the plant (Hafeez et al., 2013). Physico-chemical soil properties, mainly pH and organic matter content may influence metal sorption and, thereby, its availability and effective dose. Diffusion plays a particularly important role in the transport of Zn (as well as other nutrients, including P, K, Cu, Fe and Mn) to root surfaces in soils containing only limited quantities of plant-available Zn (Singh 2018). Zinc uptake occurs in the form of divalent cation, or as complexes involving organic ligands. At high pH levels, crops absorb Zn in the form of a monovalent (ZnOH^+^) cation (Marschner 1995). Conventional sources of this micronutrient provide Zn in ionic form to be taken up by crops. This process may involve the different micronutrient ions competing to enter the plant; as a result, what is otherwise a beneficial process of nutrient accumulation may act as a limiting factor for the efficient use of certain micronutrients (Dimkpa and Bindraban 2016). Cases of antagonism (negative interaction) have been reported between macronutrient cations such as Ca, Mg or K and the ability of plants to bind or absorb Zn (Bell et al., 1990).

In recent studies, it has been reported that plants also have the ability to take up ZnO-NPs, with the most important factor for such uptake being the size of the NPs (Lv et al., 2019; Raliya et al., 2015; Zhao et al., 2012). Different authors reported that the increased surface reactivity of nanoparticles could enlarge root pores or create new ones, leading to increased hydromineral flow in the roots (Larue et al., 2012; Raliya et al., 2015). Consequently, increased nutrient uptake and increased root length would result (Castiglione et al., 2011). Raliya et al. (2015) found that ZnO-NPs (mixture of hexagonal and quasi-spherical shapes, average geometric diameter 28 ± 0.7 nm, electrophoretic zeta potentials -29.7 ± 5.8 mV) tend to accumulate in the roots, shoots and leaves of a tomato crop. This study showed that after the initial uptake of NPs by tomato plants (whether via root or leaf cells), they were bio-distributed throughout the plant, being transported through its vascular system. The uptake and effect of metal oxide NPs on growth and metabolic functions depend on plant species, plant age, and the type of NPs used (Lv et al., 2019; Nair et al., 2010; Rico et al., 2011). Different authors have reported that high NPs concentrations induce various morpho-physiological changes in root length, shoot length, root and shoot fresh matter (FM) as well as dry matter (DM), photosynthetic attributes, and biochemical parameters (Faizan et al., 2018; Garcia-Gómez et al., 2017; Mukherjee et al., 2014; Raliya et al., 2017; Singh et al., 2016). According to Giraldo et al. (2014), NPs also have the potential to boost plant metabolism. In addition, several studies have suggested that the application of metal-based NPs influences the nutritional quality of food crops. However, these studies involving ZnO-NPs have been conducted with short exposure times, high doses in nonagricultural soils or foliar application of NPs (Ahmed et al., 2021; Garza-Alonso et al., 2021; Ketsira 2019).

Zuverza-Mena et al. (2015) reported that the contents of micro- and macro-elements, including B, Zn, Mn, Ca, Mg, P, and S, were significantly reduced in shoots and roots with application of Cu-based NPs. These results showed that Cu-based NPs/compounds depress the accumulation of nutrient elements in cilantro plants. However, the number of studies about the influence of ZnO-NPs application on the plant nutrients and the quality of the fruit still remains very limited. Medina-Velo et al. (2017b) revealed differences in the behavior of ZnO-NPs (10–300 nm, elongated morphologies), bulk ZnO and ZnCl_2_ in relation to the concentration of macro (P, Ca, Mg, S) and micronutrients (B, Cu, Fe, Mn) in a kidney bean crop. The main goal of the research reported here was to determine the influence of the application of commercial ZnO-NPs, for use as nanofertilizer, on yield and nutritional quality (macro- and micronutrients) in a high-value and widely consumed crop (*Solanum lycopersicum L* var. cerasiforme), depending on soil characteristics. The specific objectives of the work were to study the effect of applying commercial ZnO-NPs on i) crop yield, ii) macro- (N, P, K, Mg, S and Ca) and micro-nutrient (B, Cu, Fe, Mn, Na and Zn) concentrations in the different parts of the crop (root, stem, leaves and tomato fruits) and iii) the extent of nutrient translocation to the aerial part in tomato plants.

## 2 Materials and methods

### 2.1 Nanoparticle characterization

**Figure 1:**
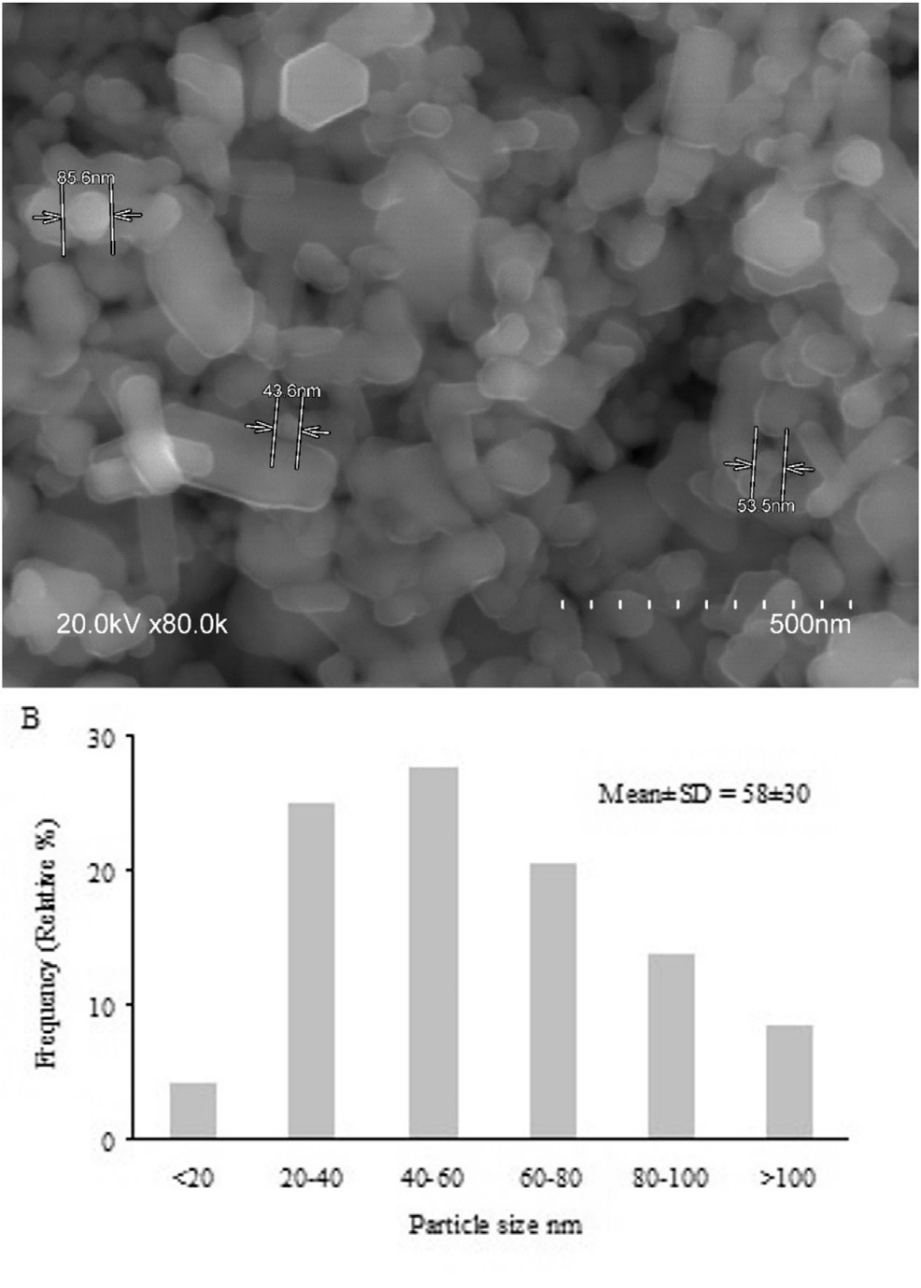
(A) SEM micrographs of ZnO NPs (B) Frequency distribution of the particle sizes (longer dimension) derived from the TEM micrographs of ZnO NPs.

The ZnO-NPs used in this study were commercially available nanoparticles obtained from Sigma-Aldrich (Germany) (primary particle size ≤50 nm; specific surface area 15–25 m^2^/g). The size and shape of the nanoparticles were previously determined by the authors using a transmission electron microscope (TEM) (Figure 1) (Almendros et al., 2020; García-Gómez et al., 2020). ZnO-NPs were rod-and elongated-shaped with a mean length (longest dimension) of 55 ± 27 nm, The zeta-potential of the zinc nanoparticles in solutions was −7.2. The average hydrodynamic diameters of aggregates were 503 ± 142 (10% intensity) and 1486 ± 244 nm (90% intensity) (García-Gómez et al., 2020).

### 2.2 Soil characterization

The soils were collected from the Ap horizon (0–20 cm) of two crop fields located in central Spain (Soil 1: 40° 44′ N, 3° 25′ W; and Soil 2: 40° 22′N, 3° 24′ W). The soils were air-dried, and fractions of less than 2 mm were used in the experiment. These soils were not sterilized, i.e. they would contain native microbes that survive the drying process. These soils were characterized using standard analytical determinations in a previous work (Garcia-Gómez et al., 2017). Both were soils commonly used to cultivate cereals and were characterized by their low organic matter contents (<2%). Both types of soil are common in the Mediterranean area. Soil 1 was classified as a TypicPalexeralf and Soil 2 as a TypicHaploxerepts (Soil Survey Staff 2014). Soil 1 was acidic (pH = 5.4), while, in contrast Soil 2 was calcareous (pH = 8.5). The soils were classified as Luvisol and Cambisol (FAO 2015).

The general soil properties, based on means from three replicates, are reported in Table 1. The total Fe, Cu, Mn and Zn concentrations were higher in Soil 2 than in Soil 1. The DTPA–triethanolamine (TEA)-extractable Fe, Cu, Mn and Zn were higher in Soil 1 than in Soil 2. Zinc concentration in Soil 1 is adequate, Soil 2 is Zn-deficient, with DTPA-Zn < 0.5 mg kg^−1^ (Jones 2001).

**Table 1:** Main physicochemical parameters and element concentrations measured in acidic and calcareous soils.

	Acidic soil	Calcareous soil
pHw (1:2.5 w:v)	5.5	8.5
Classification (FAO 2015)	Luvisol	Cambisol
Texture (USDA)	Silt loam	Silty clay loam
Sand (g kg^−1^)	250	175
Silt (g kg^−1^)	570	435
Clay (g kg^−1^)	180	390
Organic matter (g kg^−1^)	16.9	11.3
Total carbonate (g kg^−1^)	Nd	106
Free carbonate (g kg^−1^)	Nd	24
EC (μS cm-1) (1:5 w:v)	66.9	125.9
Available P (mg kg^−1^)	10.7	16.1
Base saturation (g kg^−1^)	54.7	100
CEC (cmol kg^−1^)	11.4	22.1
Total N (g kg^−1^)	0.91	0.9
C:N ratio	13.3	10.9
Total Ca (g kg^−1^)	1.0	16.8
Total K (g kg^−1^)	6.6	17.5
Total Mg (g kg^−1^)	2.2	58.9
Total P (g kg^−1^)	0.2	0.9
Total S (g kg^−1^)	0.1	0.4
Total B (mg kg^−1^)	6.54	26.25
Total Cu (mg kg^−1^)	9.6	24.5
Total Fe (mg kg^−1^)	14515	14600
Total Mn (mg kg^−1^)	518	162
Total Na (mg kg^−1^)	300	800
Total Zn (mg kg^−1^)	40	62
DTPA-TEA-Extractable Metal (mg kg^−1^):		
Fe	31.9	4.42
Cu	0.88	0.99
Mn	105.30	27.84
Zn	2.49	0.28

E.C. electrical conductivity, CEC cation exchange capacity.

### 2.3 Greenhouse pot experiment

Pots in which different rates of ZnO-NPs were applied to the two soils (acidic and calcareous) were placed in an uncontrolled greenhouse environment on the Universidad Politécnica de Madrid campus, under environmentally realistic conditions. Cherry tomato (*Solanum lycopersicum* L var. cerasiforme.) plants were grown in polyethylene pots (10 L capacity, 24 cm mean internal diameter and 24 cm height). 10 kg of air-dried soil was used. To facilitate aeration and drainage, a 1.5 cm thick layer of washed gravel and polyester mesh was placed at the bottom of the pot. The potting soil was fertilized with NPK (100 mg N kg^−1^ as urea, 50 mg P kg^−1^ as KH_2_PO_4_ and 125 mg K kg^−1^ as KH_2_PO_4_ and K_2_SO_4_). The different Zn treatments were then applied to the NPK-fertilized soils.

Different ZnO-NPs application rates were tested: a low rate (3 mg Zn kg^−1^), a medium rate (20 mg Zn kg^−1^) and a high rate (225 mg Zn kg^−1^). The low rate was tested to reflect ZnO-NPs concentrations that can be found naturally in soils (Tiede et al., 2009). The medium rate was selected because experimental studies suggest that this concentration of ZnO-NPs is usually beneficial to plants (Reddy Pullagurala et al., 2018). The concentration of 225 mg Zn kg^−1^ was chosen to evaluate possible inhibitory effects due to a high ZnO NPs concentration (above 200 mg Zn kg^−1^) (Zuverza-Mena et al., 2017). Zinc application was performed in the top 8 cm of soil to simulate spreading and mixing in the topsoil layer. Therefore, total Zn concentration values in the top soil layer (0–8 cm) were initially higher (10:3 ratio) than the average Zn application rate in the pot. A ZnO-NPs non-treated group, named Nil-ZnONP, was used as a control in each of the soils. Nil-ZnONP refers to ZnO-NPs-free soils, although in both soils background levels of natural Zn are present but bioavailability very different (Table 1). Three replicates were used for each treatment, with a total of 24 pots in a randomized arrangement. After 3 days following soil treatments with ZnO-NPs, 10 cherry tomato seeds were sown in the top layer of soil in each pot. Three cherry tomato plants were grown along 3 months in the containers from seed until tomato fruits were produced. Temperatures ranged from 4 °C (night) to 38 °C (day) and relative humidity from 20% to 85%. The soils were irrigated with tap water during the experiment. The main physicochemical parameters of tap water were: pH, 7,5; conductivity, 132 μS cm^−1^; NH_4_+, 0,24 mg L^−1^; NO_3_^-^, 2 mgL^-1^, NO_2_^-^, ˂0,1 mg L^−1^; free chlorine residual, ˂0.15 mg L^−1^; chlorine, 14 mg L^−1^; SO_4_^2-^, 4 mg L^−1^; calcium, 9 mg L^−1^, magnesium, 1,4 mg L^−1^; sodium, 7 mg L^−1^, potassium, 0.8 mg L^−1^; fluorine, ˂ 0.1 mg L^−1^, aluminum, 20 μg L^−1^; iron, ˂50 μg L^−1^; manganese, ˂20 mg L^−1^; boron, ˂0.2 mg L^−1^; copper, ˂ 0,05 mg L^−1^. The cherry tomato fruits were gradually harvested. After this, they were washed in deionized water and vacuum freeze-dried.

Plants were harvested and washed in deionized water. The different vegetative parts (roots, stems and leaves) were separated, and their fresh weights were recorded. To desorb Zn from root surfaces, they were first washed in deionized water in an ultrasound-assisted bath (35 kHz, 15 min) and then in Na_4_EDTA (10 mmol L^−1^, 15 min) in the ultrasound-assisted bath (Zhou et al., 2011). By following this procedure, it is possible to know the uptake of Zn by the root and to exclude the concentration of Zn that is strongly adsorbed on the surface of plant roots, and part of it by mechanical adhesion. The adsorption of nanoparticles on the root surface is often mistakenly considered as their uptake (Zhou et al., 2011). The procedure used to exclude the concentration of Zn that is strongly adsorbed on the root surface of the plants resulted in desorption of all nutrients found on the root surface. These nutrient concentrations were not measured. The concentration of desorbed nutrients would also provide insight into particle binding of Al and Si levels.

Finally, all plant samples were dried in a forced draft oven at 60 °C until a constant weight was reached.

### 2.4 Nutrient contents in plant

The total C and N contents in the different parts of the tomato plants (fruit, leaf, stem, and root) were determined by elemental analysis with a LECO TruMac CN analyzer®. Concentrations of P, K, Mg, S, Ca and microelements (B, Cu, Fe, Mn, Na and Zn) in the plant samples were measured using 0.05–0.2 g of dry tissue sample. These samples were digested (220 °C, 340 bar) with a HNO_3_:H_2_O_2_ (4:1, v/v) solution in a microwave oven (Milestone Ethos). Digested samples were analyzed by using inductively coupled plasma spectrometry (ICP-OES, Thermo iCAP Dual 6500).

The root-shoot translocation factor (TF) was used to study the extent of nutrient translocation within the plant, from inside the root to the shoot. This factor was calculated considering the nutrient concentrations and weights of each of the plant parts (tomato fruits, leaves and stem) in order to estimate the average concentration in the aerial part.

### 2.5 Daily dietary intake of nutrients

The human daily dietary intake (DDI) and recommended nutrient intake (mg d^−1^) of macro- and micro-nutrients were according to Khan et al. (2016). The DDI of each nutrient was determined on fresh weight basis and was calculated on individual body weight basis:

DDI = (nutrient concentration × daily food intake)/body average weight

The projected dietary intakes for adults and children were compared with recommended dietary allowance (RDA) values for each nutrient and presented as percentage of the RDA (Higdon 2001; Khan et al., 2016).

### 2.6 Statistical analysis

A correlation analysis and statistical analyses were made using Statgraphics Centurion XVII 17.2 software (Manugistic, Rockville, MD). Analysis of variance (ANOVA) of the different parameters was performed. The main effects of ZnO-NPs rate were differentiated using Fisher's LSD test at a probability level of P ≤ 5%. Different orthogonal contrasts were used to compare the effects of added ZnO-NPs on the macro- (N, P, K, Mg, S and Ca) and micro-nutrient (B, Cu, Fe, Mn, Na and Zn) concentrations. Multifactor ANOVAs) of the studied TFs were carried out to determine the main effects of the ZnO-NPs application rate, soil type and experimental repetition, and the interactions between them. When the two-way interaction between Zn rate and N rate factors was significant, we performed a new multifactor ANOVA to determine the main effects of the combined factor. A principal component analysis (PCA) biplot was constructed which considered the concentration of macro- and micro-nutrients in the different plant parts (root, stem, leaf and fruits).

## 3 Results

### 3.1 Yield in tomato plants

**Figure 2:**
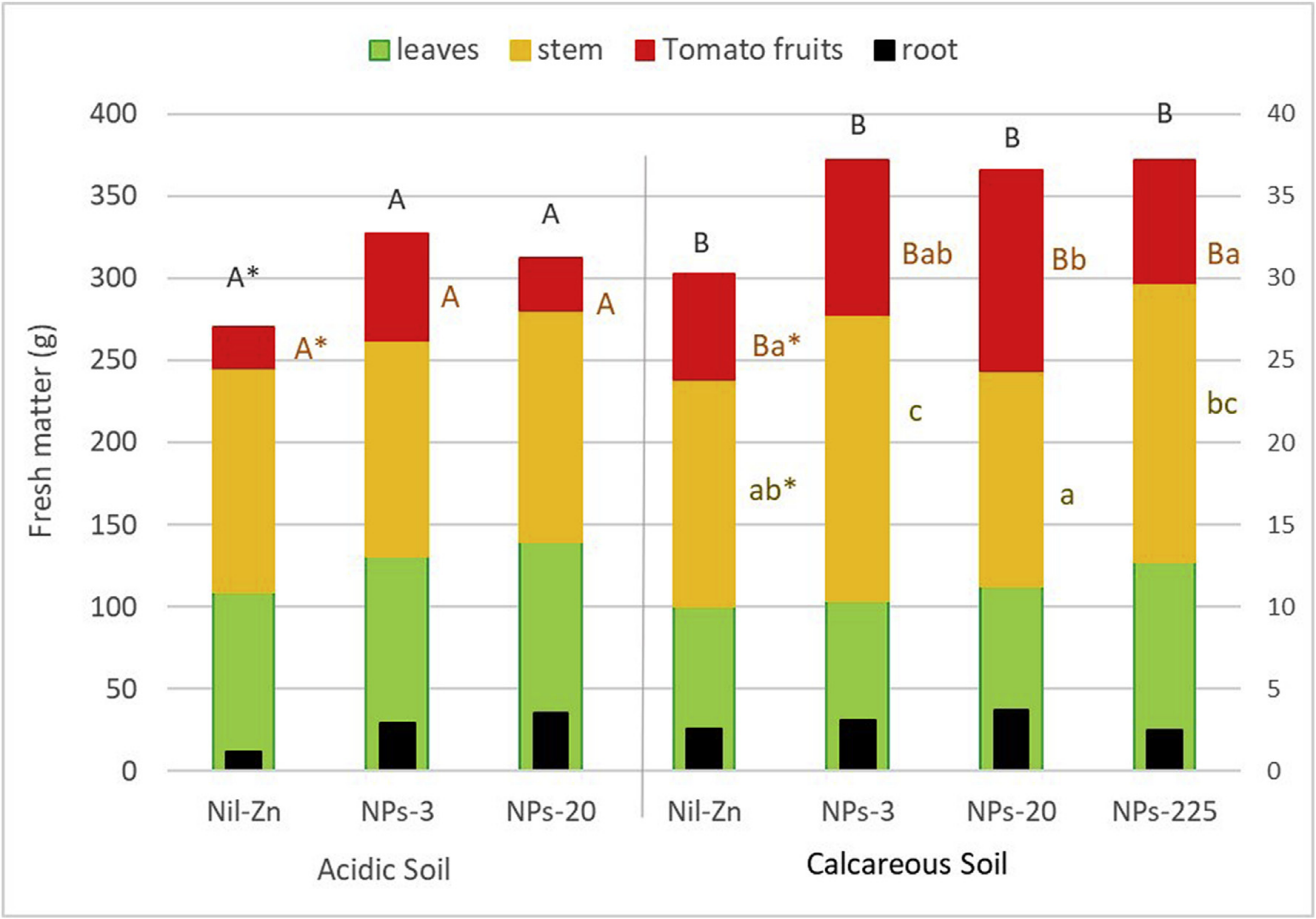
Effect of ZnO-NPs on fruit yield and plant biomass (fresh matter) of tomato plants grown in two different soils. The scale of the secondary Y-axis reflects the root fresh matter. Statistically significant differences at P < 0.05 (LSD test) are presented by different letters. The letter above the bar indicates significant differences for total fresh matter (FM). Capital and small letters indicate the differences for each part of the plant between soils and ZnO-NPs rate, respectively.

Fresh matter (FM) yields of the different plant parts (root, stem, leaves, and fruit) are shown in Figure 2. In the acidic soil, the highest dose of ZnO-NPs (225 mg Zn kg^−1^ soil) inhibited growth of the cherry tomato plants, causing their death. Interestingly, extensive root development of the tomato plants was obtained in both soils, with root distribution throughout the pot but especially at depth. This effect can be explained by the effect of the concentration and gradient of NPs in the pots, i.e., the effect of the top layer. This root development influences the absorption of nutrients due to the surface area (Machado and Oliveira, 2005). However, despite the extent of root development, the influence to the total fresh biomass of the plant was low.

In the acidic soil, FM yields did not show significant differences between the different treatments. Plant yield grown in this acidic soil amended with 3 mg kg^−1^ (ZnO-NPs-3) reached values of between 1.0 and 2.6 times (stem and tomato fruit, respectively) the yield obtained with the Nil-ZnO NP treatment. There was a trend towards an increase in stem and leaf FM with respect to ZnO-NPs-3 when the ZnO-NPs-20 treatment was applied (7% increase in both leaf FM and stem FM). This trend was reversed in tomato fruits, with yield decreasing by as much as 52% with the ZnO-NPs-20 treatment with respect to the ZnO-NPs-3.

In the calcareous soil, the application of ZnO-NPs significantly (P < 0.05) increased total FM yields, reaching increases of 21%–23% with respect to the Nil-ZnO NP treatment. In this soil the application of ZnO-NPs at the rate of 20 mg Zn kg^−1^ significantly (P < 0.05) increased the FM yields of tomato fruits, reaching increases of 91% with respect to Nil-ZnNP. However, when the highest rate of ZnO-NPs (225 mg Zn kg^−1^) was applied in this calcareous soil, tomato fruit FM yields decreased, approaching values close to those of the Nil-ZnO NP treatment. The application of ZnO-NPs at the rate of 20 mg Zn kg^−1^ produced the lowest FM stem yield (P < 0.05), although no significant differences were obtained with respect to the Nil-ZnO NP treatment. It is noteworthy that the FM yield values obtained for root, stem and tomato fruit in the calcareous soil were higher than those achieved in the acidic soil, for the same application rate of ZnO-NPs. This difference between acidic and calcareous soil was significant for total plant FM (P < 0.05) and for tomato fruit FM (P < 0.05) (Figure 2).

### 3.2 Macronutrient (N, P, K, Mg, S and Ca) and micronutrient (B, Cu, Fe, Mn, Na and Zn) concentrations in tomato plants

The factorial ANOVA and orthogonal contrasts on the concentration of macronutrients and micronutrients in tomato plant, root, stem, leaves and tomato fruits are shown in Tables 2 and 3, for the acidic and calcareous soil, respectively.

**Table 2:** Macro and micronutrients concentrations in fruit, root, stem, leaves and total plant obtained with the different ZnO-NPs rates in the acidic soil. Values compared using LSD multiple-range test at the 0.05 level of probability. Homogeneous groups are denoted with the same letter. Italic values show significant orthogonal contrast at the 0.05 level of probability between treatment means (Nil- ZnO-NPs *vs* ZnO-NPs) and the estimated difference between them. ∗∗∗, ∗∗, and ∗ denote significance at 0.0001, 0.001, and 0.05, respectively.

	ACIDIC SOIL														
	Treatment	tomato fruits		root			stem			leaves			total		
Macronutrients concentration ​mg 100g plant^−1^															
N	Nil-ZnNP	350.65		898.27			148.07			399.49			273.74		
	NPs-3	373.85		696.37			167.41			383.08			244.90		
	NPs-20	249.07		781.96			141.38			432.76			293.47		
Ca	Nil-ZnNP	10.17		229.49			69.87			169.08			107.12		
	NPs-3	19.09		143.27			98.21			158.88			91.55		
	NPs-20	10.90		159.69			75.24			160.54			107.85		
K	Nil-ZnNP	338.14		467.55			500.31			504.73	b∗	*0.10∗*	502.84		
	NPs-3	296.60		490.26			545.71			431.14	a		495.54		
	NPs-20	362.12		530.65			540.32			477.93	ab		501.03		
Mg	Nil-ZnNP	15.95		64.74			14.06			49.33			28.80		
	NPs-3	21.12		58.75			21.27			49.37			29.53		
	NPs-20	20.68		53.42			14.52			42.18			28.43		
P	Nil-ZnNP	46.02		47.87			30.79			40.46			39.28		
	NPs-3	45.10		28.24			20.04			30.86			29.70		
	NPs-20	59.11		42.19			32.13			29.45			34.90		
S	Nil-ZnNP	15.48		54.20			26.16			165.83			81.19		
	NPs-3	17.48		45.57			27.15			162.91			62.27		
	NPs-20	18.18		52.61			20.60			132.37			72.36		
Micronutrients concentrations mg∙kg plant^−1^															
B	Nil-ZnNP	0.61		8.21	*6.08∗*		1.38			5.58			2.87		
	NPs-3	1.15	*1.71∗*	5.15			1.58			4.45			2.13		
	NPs-20	0.71		5.19			1.62			5.00			3.11		
Cu	Nil-ZnNP	0.92		2.86			0.41			1.34			0.83		
	NPs-3	0.73		2.89			0.50			1.45			0.78		
	NPs-20	0.99		3.09			0.52			1.14			0.88		
Fe	Nil-ZnNP	5.47		644.75			4.70			22.28			12.46		
	NPs-3	6.61		1586.97			11.31			18.46			14.49		
	NPs-20	4.94		1336.24			6.20			14.42			14.54		
Mn	Nil-ZnNP	6.39		54.74			18.34			91.16			44.35		
	NPs-3	10.07		76.59			37.71			124.13			55.15		
	NPs-20	8.20		120.08			27.13			99.67			59.14		
Na	Nil-ZnNP	0.22		30.34	b∗	*29.08∗*	0.83			1.22			0.90	a∗	
	NPs-3	0.30		15.42	a		2.80			2.81			2.78	b	
	NPs-20	0.25		16.17	a		0.76			1.12			0.93	a	
Zn	Nil-ZnNP	3.61		54.76			11.16	a∗		4.83	a∗∗∗		8.12	a ∗	
	NPs-3	3.55		55.11			18.80	a	*15.52∗∗*	6.25	b	*9.90∗*	13.43	b	*14.58∗*
	NPs-20	4.78		137.98			28.06	b		13.30	c		19.05	c

**Table 3:** Macro and micronutrients concentrations in fruit, root, stem, leaves and total plant obtained with the different ZnO-NPs rates in the calcareous soil. Values compared using LSD multiple-range test at the 0.05 level of probability. Homogeneous groups are denoted with the same letter. Italic values show significant orthogonal contrast at the 0.05 level of probability between treatment means (nil- ZnO-NPs *vs* ZnO-NPs) and the estimated difference between them. ∗∗∗, ∗∗, and ∗ denote significance at 0.0001, 0.001, and 0.05, respectively.

	CALCAREOUS SOIL											
	Treatment	tomato fruits			root			stem			leaves	total
Macronutrients concentration mg 100g plant^−1^												
N	Nil-ZnNP	210.61			646.81			84.17			226.31	172.42
	NPs-3	200.30			595.70			68.06			173.86	140.13
	NPs-20	185.01			502.31			80.22			125.40	140.15
	NPs-225	227.88			675.43			86.53			205.39	145.50
Ca	Nil-ZnNP	8.66			315.54			60.15			224.83	100.37
	NPs-3	7.92			260.61			68.32			203.08	87.54
	NPs-20	8.22			295.80			59.76			285.25	92.31
	NPs-225	6.95			272.04			51.58			226.32	108.84
K	Nil-ZnNP	400.72			404.04			545.11			310.14	461.84
	NPs-3	417.62			450.95			429.29			339.41	431.26
	NPs-20	411.68			479.44			461.10			238.46	388.50
	NPs-225	378.32			432.64			538.19			386.32	465.84
Mg	Nil-ZnNP	26.28	c ∗	*0.01∗*	587.85			69.02			183.48	96.02
	NPs-3	24.08	bc		463.55			52.91			144.65	70.68
	NPs-20	21.05	ab		616.04			70.36			150.57	73.98
	NPs-225	19.46	a		412.02			59.35			156.35	87.61
P	Nil-ZnNP	64.88		*0.03∗*	41.92			45.46	c∗	*0.04∗*	52.77	55.09
	NPs-3	59.40			39.35			28.74	a		41.04	42.93
	NPs-20	55.28			38.46			36.66	b		46.04	47.30
	NPs-225	50.71			38.20			34.09	ab		39.06	40.78
S	Nil-ZnNP	17.63			41.74			30.90			193.43	80.34
	NPs-3	16.83			44.15			27.37			179.79	66.53
	NPs-20	16.12			38.46			32.02			171.51	68.45
	NPs-225	15.30			40.77			31.92			191.16	87.05
Micronutrients concentrations mg∙kg plant^−1^												
B	Nil-ZnNP	0.94			8.87			1.85			10.17	4.34
	NPs-3	0.85			8.52			1.58			10.56	3.80
	NPs-20	0.93			8.77			1.90			9.42	3.89
	NPs-225	0.74			8.21			1.74			9.64	4.44
Cu	Nil-ZnNP	0.98		*0.38∗*	3.58			0.55	b∗		1.59	1.03
	NPs-3	0.89			3.89			0.33	a		1.23	0.77
	NPs-20	0.78			4.33			0.50	b		1.90	0.95
	NPs-225	0.86			3.27			0.43	ab		1.71	1.03
Fe	Nil-ZnNP	3.93			2737.07			6.07			14.16	13.64
	NPs-3	3.13			2130.99			6.64			18.69	12.03
	NPs-20	3.60			3009.22			5.46			13.56	13.29
	NPs-225	3.17			1940.31			5.36			19.35	12.57
Mn	Nil-ZnNP	2.23		*1.35∗*	58.54			3.49			8.00	4.76
	NPs-3	1.98			46.53			2.82			7.91	4.07
	NPs-20	1.60			60.81			3.71			6.91	3.96
	NPs-225	1.77			45.55			3.07			7.97	4.70
Na	Nil-ZnNP	0.23			21.75			0.55			0.42	0.46
	NPs-3	0.23			19.69			0.33			0.43	0.37
	NPs-20	0.20			17.55			0.47			0.34	0.37
	NPs-225	0.21			22.18			0.48			0.48	0.47
Zn	Nil-ZnNP	2.71	ab∗∗		19.24	a∗∗	*44.58∗*	3.55	a∗∗∗	*10.06∗*	2.79	3.23
	NPs-3	2.52	a		17.91	a		3.65	a		2.93	3.29
	NPs-20	2.84	b		35.31	b		6.61	b		2.92	4.13
	NPs-225	3.49	c		49.08	c		10.44	c		4.36	7.01

In the acidic soil, as expected all Zn treatments numerically increased Zn concentration in the different parts of the tomato plants. However, these increases were only significant in stem (P < 0.05), leaves (P < 0.0001) and total plant Zn concentration (P < 0.05) (Table 2). The orthogonal contrast used to compare the effect of Nil-ZnO NP *vs* Zn treatments also showed differences in stem (P < 0.001), leaves (P < 0.05) and total plant Zn concentration (P < 0.05).

In this acidic soil, there were no significant differences between the ZnO-NPs treatments in the concentrations of N, Ca, Mg, P and S in the different parts of the tomato plants (Table 2). The orthogonal contrast used to compare the effect of Nil-ZnO NP *vs* ZnO-NPs treatments only showed differences (P < 0.05) in K concentration in leaves. The estimated difference between treatment means was lower when ZnO-NPs were applied. The application of ZnO-NPs at the rate of 3 mg Zn kg^−1^ produced the lowest K concentration in leaves, with a 14% decrease compared to the Nil-ZnO NP treatment.

Differences (P < 0.05) were also obtained between Nil-ZnO NP and Zn treatments for B concentration in tomato fruits and also for B and Na concentration in the root. These estimated differences between treatment means were only greater for the NPs treatments than for Nil-ZnO NP for B concentration in tomato fruits. The application of ZnO-NPs decreased the Na concentration in tomato roots by 47–49% compared to the Nil-ZnO NP treatment.

In the calcareous soil, all Zn treatments numerically increased the Zn concentration in the different parts of the tomato plants. These increases were statistically significant in tomato fruits (P < 0.001), root (P < 0.001), and stem Zn concentration (P < 0.0001) (Table 3). Application of ZnO-NPs and increasing the application rate produced decreases in Mg concentration in tomato fruits with respect to the Nil-ZnO NP treatment (6%, 18% and 25% for NPs-3, NPs-20 and NPs-225, respectively). As shown in Table 3, the orthogonal contrast used to compare the effect of Nil-ZnO NP *vs* ZnO-NPs treatments showed differences (P < 0.05) in the concentration of Mg, P, Cu and Mn in tomato fruits. In all cases, the estimated difference between treatment means was lower when ZnO-NPs were applied.

The concentration of P and Cu in the stem decreased with the application of ZnO-NPs at 3 mg Zn kg^−1^, with respect to the Nil-ZnO NP treatment (37% and 39%, for the concentration of P and Cu in the stem, respectively). These concentrations of both nutrients increased with higher ZnO-NPs application rates, reaching increases of 28% and 51% with the application of NPs-20, with respect to NPs-3, for P and Cu concentration in the stem, respectively.

Orthogonal contrasts were also used to compare the effect on elements content of the highest rate of ZnO-NPs (225 mg Zn kg^−1^) *vs* other treatments in this calcareous soil. Significant differences (P < 0.05) between treatment means were obtained for Zn concentration in all plant parts (estimated difference: 2.41, 74.78, 17.51, 4.49 and 10.39 for tomato fruits, root, stem, leaves, and total, respectively), Mg concentration in tomato fruits and K concentration in leaves (estimated difference: 0.01, and 0.27, respectively). The estimated difference between treatment means was greater for the ZnO-NPs-225 treatment in Zn concentration in all plant parts and in K concentration in leaves. In contrast, the estimated difference between treatment means in Mg concentration in tomato fruits was lower for the NPs-225 treatment.

The orthogonal contrast used to compare the effect of Nil-ZnO NP and NPs-3 *vs* NPs-20 and NPs-225 showed significant differences (P < 0.05) in fruit, root, stem and total Zn concentration in tomato plant in this calcareous soil. The estimated differences between the means of the treatments (NPs-20 + NPs-225 *vs* Nil-ZnO NP + NPs-3) were 1.10, 47.25, 9.85 and 4.62, respectively. Orthogonal contrasts also showed statistically significant differences (P < 0.05) between the means of these treatments (Nil-ZnO NP + NPs-3 *vs* NPs-20 + NPs-225) for Mg, P and Mn concentration in tomato fruits (estimated difference: 0.01, 0.02 and 0.84, respectively). Although the estimated difference between treatment means was the highest for NPs-20+NPs-225 for Zn concentrations in tomato fruits, it was the lowest for Mg, P and Mn concentrations.

**Figure 3:**
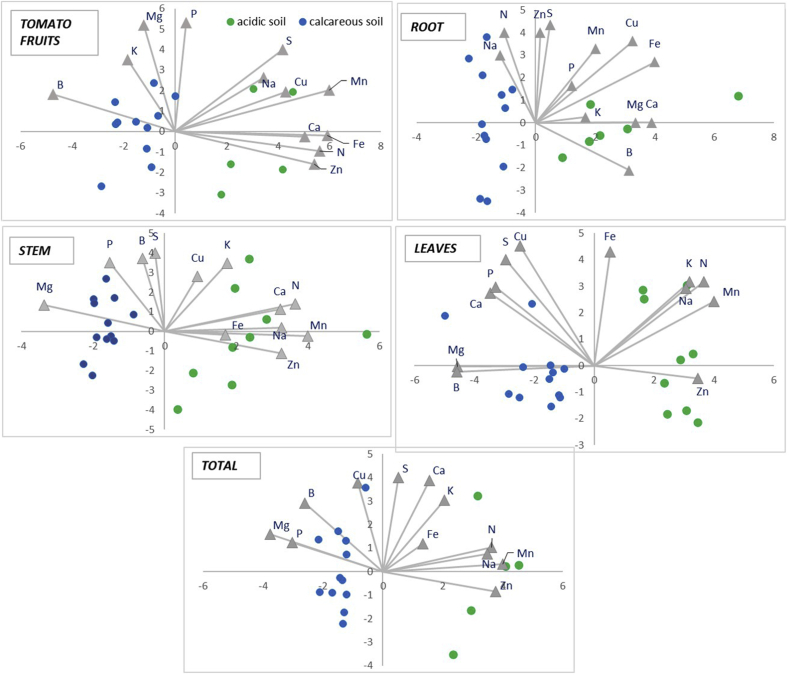
Biplot of the first two principal components of the principal component analysis (PCA) of nutrient concentration in the different plant tissues (dry matter).

To study the possible relationships of the different nutrients, a PCA biplot was generated. The PCA analysis reveals variables affecting macro- and micronutrients in dry weight for either of the soils (Figure 3). The variance explained by the first two components was 73.61% for tomato fruits, 76.97% for root, 70.57% for stem, 75.02% for leaves, and 74.76% for total plant. The biplot of the first two components shows different groups of values according to soil type: calcareous soil on the left on the x-axis and acid soil on the right on the x-axis. The different application rates are grouped together and cannot be differentiated, which suggests that nutrient availability reaches similar levels in each soil and/or that the plant evens out the effect of different doses.

According to the PCA biplot for tomato fruits, the concentrations of Zn, N, Fe and Ca are quite near to each other, indicating a close and positive relationship between them. This suggests that these nutrients had similar behavior in tomato fruits. In contrast, the straight angle shown in the PCA biplot between Zn and B concentrations reveals an opposite relationship between them. The right angle between Zn and P concentrations indicates that these concentrations are not related.

The PCA biplot obtained with the nutrient concentration in the root revealed a positive relationship between Zn and S. In contrast, none of the K, Mg and Ca concentrations showed any relationship with the Zn concentration in the root. Regarding nutrient concentration in the stem, the PCA biplot revealed a positive relationship between Zn, Na, Mn, Fe, N and Ca concentrations. The PCA biplot for stem and leaves showed an opposite relationship between Mg and Zn concentrations. The PCA biplot obtained with nutrient concentration in leaves also revealed an opposite relationship between Zn and Mg or B concentrations in this part of the tomato plant.

The PCA biplot for total nutrient concentrations in tomato plants showed a close and positive relationship between Zn, N, Mn and Na concentrations. In contrast, total S concentrations showed no relationship with total Zn concentration. This PCA biplot also revealed an opposite relationship between Zn and Mg or P concentrations in the tomato plants.

### 3.3 Translocation of nutrients in tomato plants

Several authors have studied nutrient mobility within plants by calculating TFs (Zhang et al., 2019). When TF is calculated as the ratio between the nutrient concentration in the shoot of the plant and nutrient concentration in the root of the plant, a high TF value indicates high nutrient mobility and the possibility of accumulating large amounts of nutrients in the aerial part of plants (Alloway 2013; Almendros et al., 2019; Intawongse and Dean 2006).

**Figure 4:**
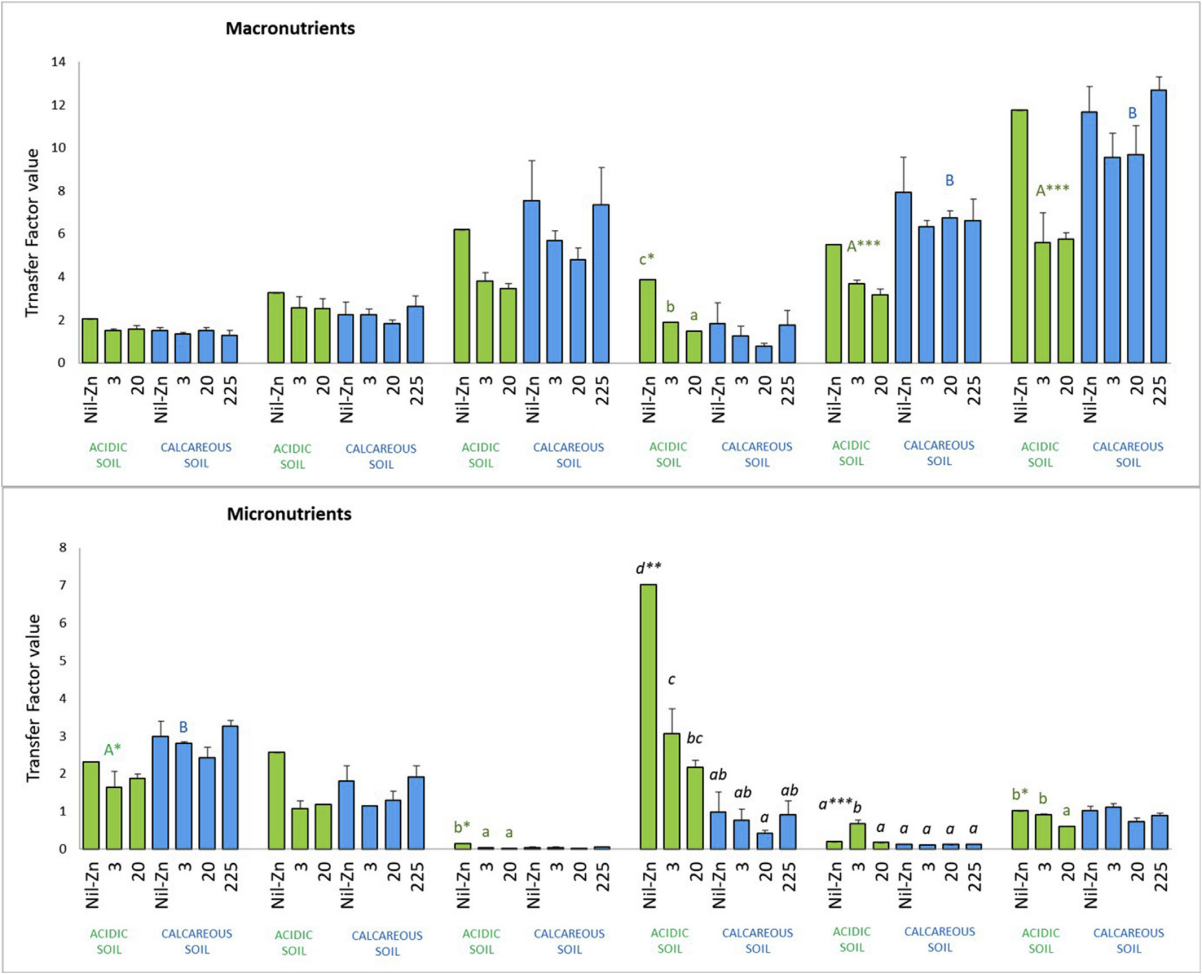
Translocation factors (TF) in both soils treated with different ZnO-NPs rates (3, 20 and 225 mg Zn kg^−1^). Statistical differences at P < 0.05 (LSD test) are presented by different letters, according to the statistical interactions obtained. Capital and small letters indicate the differences between soils and ZnO-NPs rates, respectively. Letters in italics indicate statistically significant differences between treatments for the combined factor of soil x ZnO-NPs rate. ∗∗∗, ∗∗, and ∗ denote significance at 0.0001, 0.001 and 0.05, respectively.

As can be seen in Figure 4, the influence of the different ZnO-NPs treatments applied to the soils on element distribution within the tomato plant varied according to the macronutrient or micronutrient studied. In the case of TF-P, TF-S and TF-B, statistically significant differences were obtained between acidic and calcareous soil. The mean values of TF-P, TF-S and TF-B were higher in the calcareous soil than in the acidic soil (1.7, 1.4 and 1.5 times, respectively). Contrary to these nutrient TFs, the mean value of TF-Mn and TF-Na were 5.4 and 3.0 times higher in the acidic than in the calcareous soil. However, the combined factor soil × Zn application rate significantly affected TF-Mn and TF-Na. TF values for these nutrients increased up to 17.0 (TF-Mn) or 6.3 (TF-Na) times depending on treatment and soil. TF-Mn was lower in calcareous soil than in acidic soil, except for the ZnO-NPs-20 treatment in acidic soil which was statistically equal to that obtained with Nil-ZnNP, ZnO-NPs-3 and ZnO-NPs-225 in calcareous soil. The highest TF-Mn was obtained with the Nil-ZnO NP treatment in calcareous soil. The highest TF-Na was obtained with the ZnO-NPs-3 treatment in acidic soil. In the case of TFs-Mg, TF-Fe and TF-Zn, statistically significant differences were obtained between treatments in the acidic soil. These values ranged from 1.5 to 3.9, from 0.02 to 0.15 and from 0.6 to 1.0 (for TFs-Mg, TF-Fe and TF-Zn; ZnO-NPs-20 and Nil-ZnO NP treatments, respectively).

## 4 Discussion

Our results demonstrate that ZnO-NPs used as nanofertilizers could be an effective strategy to increase tomato crop yield. In the acidic soil, the yield of the edible part of the tomato plant amended with ZnO-NPs-3 was approximately two and a half times higher than with Nil-ZnNP. However, with the application of 20 mg Zn kg^−1^ the yield approached values close to the Nil-ZnO NP treatment. In the calcareous soil, tomato fruit yields were approximately twice as high with ZnO-NPs-20 than with Nil-ZnNP. However, with the application of 225 mg Zn kg^−1^ the yield approached values close to the Nil-ZnO NP treatment. These results suggest that ZnO-NPs-20 in the acid soil and ZnO-NPs-225 in the calcareous soil produced an overdose, decreasing tomato fruit yield. Different authors (Palacio-Márquez et al., 2021; Raliya et al., 2015) have reported that exposure to nanoparticles induced plant growth and development at low concentrations but decreased them at high concentrations, which could be due to bioavailable Zn toxicity. Several studies show that nanoparticles enhance biomass, produced relatively more fruit and boost plant growth in the critical growth period, but the mechanism underlying the increase in plant biomass has not yet been determined (Ketsira 2019; Kole et al., 2013; Raliya et al., 2015; Rico et al., 2014).

The FM plant yield increased by up to 16% in the acidic soil and 23% in the calcareous one with the application of ZnO-NPs with respect to the Nil-ZnO NP treatment. However, the excessive application rate (225 mg kg^−1^) decreased tomato yield. Raliya et al. (2015) conducted an experiment with a tomato crop and different rates of ZnO-NPs (from 0 to 1000 mg Zn kg^−1^). They reported that the highest biomass yield in the tomato crop was achieved with ZnO-NPs at the rate of 100 mg Zn kg^−1^, showing an increase of 40.7% over the control. Different authors (Garcia-Gómez et al., 2017; Olayinka et al., 2021; Raliya et al., 2015) consider that the increase in biomass content could be correlated with chlorophyll content and carbonic anhydrase activity, as increased light absorption by plant leaves could ultimately lead to higher biomass. Zinc acts as a structural and catalytic component of proteins and enzymes and as a co-factor for normal development of pigment biosynthesis (Balashouri and Prameeladevi 1995). Faizan et al., (2018) reported that presence of ZnO-NPs in tomato plants enhanced antioxidant systems and accelerated proline accumulation that could provide plant stability and improve photosynthetic efficiency.

**Figure 5:**
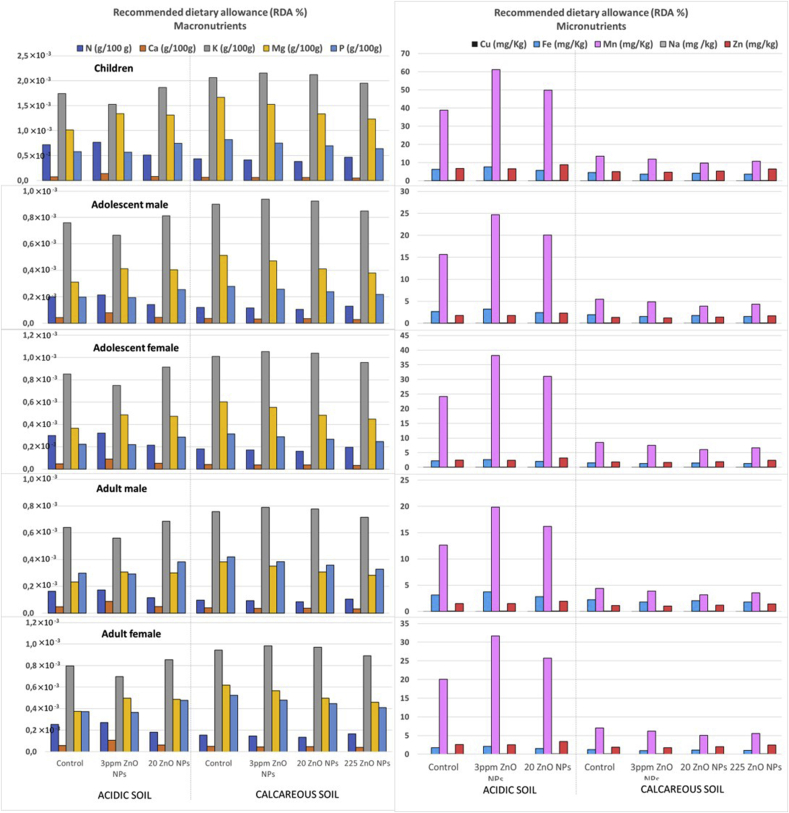
Daily dietary intake values (mg kg^−1^ d^−1^) of nutrients expressed as percentage of recommended dietary allowance (RDA %). The different age ranges studied include: children (<14 y), adolescents (14–18 y) and adults (>18 y).

Nutrient concentrations in plant tissues are frequently used to evaluate the nutritional quality of plants (Gonzalez et al., 2019a; Machado et al., 2018, 2020; Medina-Velo et al., 2017a). Our results show that the concentrations of macronutrients N, P, K and Mg in tomato fruits grown in both soils can be considered adequate in terms of nutritional requirements (≥0.16, 0.027, 0.29 and 0.01 g 100g^−1^, respectively) (Moreiras et al., 2003; Quemada Saenz-Badillos et al., 2016). It should be noted that the nutrient concentrations in tomato fruits were much higher than sufficient in different cases. Tomatoes grown in the acidic soil with 3 mg Zn kg^−1^ as ZnO-NPs reached a concentration of up to 2.3 times the sufficient N concentration in fruit. Likewise, the P concentration in tomato fruits reached values of 1.7–2.2 times (in acidic soil) and 1.9–2.4 times (in calcareous soil) the required concentration. The K concentrations in the different parts of the plant indicate that the characteristics of calcareous soil favor the uptake of this nutrient, regardless of the treatment used,. Tomatoes grown in this soil with Nil-ZnNP, 3 and 20 mg Zn kg^−1^ applied from ZnO-NPs reached K concentrations up to 1.4 times the sufficient concentration. In addition, the Mg concentration in tomato fruits reached values of 1.6–2.1 times (in acidic soil) and 1.9–2.6 times (in calcareous soil) the required concentration. These concentrations in tomato fruits show an adequate nutritional quality of these plants although, as shown in Figure 5, the daily dietary intake value of these nutrients expressed as percentage of the recommended dietary allowance (RDA) is not significant. On the other hand, the Ca concentration in tomato fruits grown in the calcareous soil did not reach the required concentration to be considered sufficient (0.011g 100g^−1^) (Moreiras et al., 2003). However, this effect was not related to fertilization with ZnO-NPs, since it was observed in all plants grown in the calcareous soil. The TF-Ca value obtained indicates that soil type did not affect the translocation of this nutrient inside the plant. Therefore, strategies to improve bioavailability of Ca carbonate, sulphate, phosphate and hydroxides in this calcareous soil would be recommendable to obtain adequate Ca concentration in tomato fruits.

Different authors have studied the effect on plant N concentration of the application of traditional Zn sources, such as ZnO, ZnSO_4_, Zn (NO_3_)_2_, natural complexes (Zn-lignosulphonate, Zn-amino acids and Zn-gluconate) or synthetic Zn chelates as Zn-DTPA-HEDTA-EDTA to cereals (Almendros et al., 2019; Gonzalez et al., 2019b; Kutman et al., 2011). In general, the results of these studies showed positive responses in Zn and N concentrations in plants with increasing Zn application rate. Although in our study this effect was not observed with the application of ZnO-NPs to this horticultural crop, the PCA indicated a close and positive relationship between both nutrients, in root, stem, tomato fruit and in total plant concentration. Kutman et al. (2011) reported that the positive interaction between N and Zn in cereals is due to improvements in root uptake and the translocation of Zn due to the presence of N. Likewise, Zn plays an indispensable role in protein synthesis and Zn deficiency impedes this process and affects the health and productivity of plants (Almendros et al., 2019).

In contrast, P concentration in tomato fruit and stem was significantly diminished by the application of ZnO-NPs in calcareous soil. This effect was opposite to that of Zn concentrations, which as expected increased with ZnO-NPs application (except at the lowest application rate of 3 mg Zn kg^−1^). This higher P concentration in stem and fruit in the Nil-ZnO NP treatment is in agreement with Mousavi (2011), who reported that in the absence of or in low concentrations of Zn, P transport increased in the shoot and its concentration increased in the aerial part of plant. This effect could be attributable to a defect in plant cell metabolism that is related to a Zn and P imbalance, such that by increasing Zn concentration P concentrations are affected at specific positions in the cells (Mousavi et al., 2012). Our results are not in agreement with some studies performed with biologically synthesized ZnO-NPs with a spherical oblate and hexagonal structure (Raliya and Tarafdar 2013) or spherical structure (Tarafdar et al., 2014). These authors reported that biologically synthesized nanoparticles increased the activity of soil enzymes such as phytase, acid phosphatase, and alkaline phosphatase. Therefore, they enhanced the mobilization of native P in the rhizosphere, increasing P availability and its uptake by plants. These dissimilarities may be due to the different characteristics of the NPs used in those studies, in which spherical-shaped NPs of smaller size (15–30 nm) were used than in our study.

In our study, the application of ZnO-NPs showed different effects on K concentration in plants depending on the soil studied. Only in the acidic soil was K concentration in leaves significantly affected by the application of ZnO-NPs, according to the orthogonal contrast. Different authors (Bell et al., 1990) have reported antagonism (negative interaction) between macronutrient cations such as K and Zn uptake by peanut and wheat plants. In our study, no effect was observed on K concentration in the root, nor in tomato fruits, nor on K translocation (TF-K) from root to shoot in either of the two soils studied or at any dose.

Different authors (Bell et al., 1990) have reported antagonism (negative interaction) between macronutrient cations such as Mg and Zn uptake by plants. In the calcareous soil, the effect on Mg concentration in tomato fruits was significantly affected by the application of ZnO-NPs. While Zn concentrations in tomato fruits increased with the rate of ZnO-NPs, Mg concentration in tomato fruits decreased. Magnesium concentration in tomato fruits was the lowest at the highest application rate. In the acidic soil, Mg translocation (TF-Mg) was significantly influenced by the Zn rate. The Mg translocation value obtained suggests a lower mobilization from root to shoot with the application of ZnO-NPs at the rate of 20 mg Zn kg^−1^. The PCA also revealed an opposite relationship between Zn and Mg concentrations in stem, leaves and total concentration in the plant.

Although the results apparently show no influence of ZnO-NPs application on Ca and S concentration in plants grown in either soil, the PCA revealed a positive relationship between these nutrients and Zn concentrations. This analysis showed a close and positive relationship between Zn and Ca concentrations in stem and fruit. These results are not in agreement with those of Medina-Velo et al. (2017a), who reported decreases in leaf and stem Ca concentration in a kidney bean crop amended with different types of ZnO-NPs (10–300 nm and elongated morphologies). Our PCA also revealed a positive relationship between S and Zn concentration in the root. These results are in agreement with Medina-Velo et al. (2017a), who reported an increase in root S concentrations of up to 65% in a bean crop when coated (Z-COTE-HP1®) ZnO-NPs were applied. These authors argued the increase in S concentration was due to the negatively charged surface of the ZnO-NPs used in that experiment (−23.6 ± 0.9 mV). This negative charge could affect proton ATPase activity and transport channels, increasing S uptake.

Regarding micronutrient concentrations in the tomato plant, effects of ZnO-NPs application on some micronutrient concentrations were observed depending on the soil studied. In the acidic soil, although Zn concentrations in root and fruit were not significantly affected by the application of ZnO-NPs, results obtained with the orthogonal contrast showed that B concentration in tomato roots decreased with the application of ZnO-NPs. Hosseini et al. (2007) conducted an experiment on a barley crop with two Zn sources (ZnSO_4_ and ZnO) and reported higher B concentrations in non-Zn-treated plants. They suggested that Zn plays a protective role on the outer surfaces of roots and/or root cell membranes, providing a protective mechanism against excessive uptake of B. However, B concentration in tomato fruits increased with the application of ZnO-NPs. These results had no statistically significant effect on B translocation (TF-B) from roots to shoots in soils amended with ZnO-NPs, with respect to the Nil-ZnO NP treatment.

As for Fe and Na concentrations in tomato fruit, different concentrations of ZnO-NPs did not affect the concentration of these micronutrients in the fruit. However, the PCA revealed a positive relationship between these nutrients and Zn concentrations in different plant parts: Fe x Zn in tomato stem and fruit and Na x Zn in root, stem, and total plant concentration. Plants grown in the calcareous soil did not reach adequate nutritional requirements with respect to Fe concentration (6 mg kg^−1^). This could be due to the fact that Fe availability reached *high values* in the acid soil and *medium values* in the calcareous soil (Fe-DTPA-TEA concentrations between 5.1 and 250 and between 2.1 and 5 mg Fe kg soil^−1^ in acidic and calcareous soils, respectively) (Jones 2003). Solubility of Fe decreases by approximately 1000-fold for each unit increase of soil pH in the range of 4–9 (Lindsay 1979). Iron deficiency is the most common nutritional deficiency worldwide and mainly affects children (Higdon 2001). Tomato fruits grown in this soil only reached 0.5 to 0.7 times the required Fe concentration. The contribution to the RDA by Fe reached values of up to 7.7% for children; 3.2% for male adolescents, 2.7% for female adolescents, 3.7% for male adults and 2.1% for adult females (ZnO-NPs at the rate of 3 mg Zn kg^−1^) (Figure 5). On the other hand, the results showed a decrease in TF-Fe value with NP-20 treatment, compared to Nil-ZnNP in the acid soil, indicating lower Fe mobility. These results agree with those reported by Wu et al. (2022) who found that Zn stress restricts the upward translocation of Fe from root to stem. Sodium concentrations in tomato fruits were extremely high compared to the required concentration (0.003 mg kg^−1^) (Moreiras et al., 2003). According to Aranceta and Serra Manjem (2011), 85.7% of the population exceeds the recommended Na intake, due to excess NaCl consumption. Therefore, nutritional recommendations limit the consumption of this nutrient. Although statistical results in the acid soil indicated differences in root Na concentration between the means of Nil-ZnO NP and ZnO-NPs treatments (lower Na concentration in the fertilized root), no differences in fruit Na concentration were obtained.

Moreover, Cu concentration in tomato stem and fruit decreased with ZnO-NPs application compared to the Nil-ZnO NP treatment in the calcareous soil. Different authors (Gong et al., 2020; Qiu et al., 2016) have reported an antagonistic interaction between Cu^2+^ and Zn^2+^ in the soil solution due to competition between the metal ions to bind to plant roots. This effect depends on different factors such as the solubility of the sources of metals in the soil, soil properties, environmental temperature, fertilizer or plant root growth(Luo et al., 2001).

In addition, Mn concentrations in tomato fruits decreased with the application of ZnO-NPs. These results are in agreement with other studies (Imtiaz et al., 2003; Safaya 1976; Singh and Steenberg 1974) that used traditional Zn sources and found that plant Mn concentration decreased with increasing levels of Zn application. The TF-Mn values obtained indicate that Zn rates affected the translocation of this nutrient inside the plant, since the highest values in each soil were obtained with the Nil-ZnO NP treatment. In contrast, the PCA revealed a positive relationship between Mn and Zn concentrations in the stem. These results are in agreement with Medina-Velo (Medina-Velo et al., 2017a), who reported a 73% increase in stem Mn concentration in a bean crop when different ZnO-NPs were applied. Daily dietary intake values (mg kg^−1^ d^−1^) of Mn were significantly (P < 0.0001) higher in the acidic soil (Figure 5). Root exudates make an important contribution to plant uptake of soil Mn in acidic soils. In this soil, the contribution to RDA by Mn reached values of up to 61% for children; 24.7% for male adolescents, 38.1% for female adolescents, 19.9% for male adults and 31.6% for adult females (ZnO-NPs at the rate of 3 mg Zn kg^−1^). There is currently no evidence that a diet based on Mn-rich vegetables causes Mn toxicity (Higdon 2001).

As expected, the Zn concentration in tomato fruits reached values higher than those required according to Moreiras et al. (2003). This concentration was reached even in treatments in which ZnO-NPs were not applied. The lowest rate of ZnO-NPs (3 mg Zn kg^−1^) and the Nil-ZnO NP treatment only showed differences in Zn concentration in the leaves of the plants grown in the acidic soil. This low application rate reflects the ZnO-NPs concentrations that can be found naturally in soils (Tiede et al., 2009). The highest TF values reached in the acidic soil were observed with these treatments (Nil-ZnO NP and ZnO-NPs-3). Differential nutrient partitioning is a key factor in fruiting tissue production. In our study, it was observed that low Zn concentration does not affect Zn accumulation in fruits, as the selective transport of the nutrient to the reproductive organs is promoted. Our results suggest that in treatments with a very high Zn dose the root preferentially retains Zn to limit toxicity in aerial tissues.

In conclusion, this study shows that Zn applied as a nanofertilizer in the form of ZnO-NPs can be used to obtain higher crop yield and adequate Zn biofortification in cherry tomato crop. The effect of the application of ZnO NPs on both crop yield and nutrient uptake in cherry tomato depends on soil characteristics. Doses of 3 mg Zn kg^−1^ in acidic soil and 20 mg Zn kg^−1^ in calcareous soil are the optimum application rates since the application of higher doses produced decreases in tomato fruit yield. In the acidic soil, the application of ZnO-NPs increased the concentration of B in tomato fruits. In contrast, in the calcareous soil the application of ZnO-NPs decreased the concentration of Mg, P, Cu and Mn in tomato fruits. These findings also indicate that nutrient concentrations in fruits are adequate for human consumption.

## Declarations

### Author contribution statement

Patricia Almendros; Demetrio González; Ana Obrador: Conceived and designed the experiments; Performed the experiments; Analyzed and interpreted the data; Contributed reagents, materials, analysis tools or data; Wrote the paper.

María Dolores Fernández; Concepción García-Gomez: Conceived and designed the experiments; Contributed reagents, materials, analysis tools or data; Wrote the paper.

### Funding statement

This work was supported by the Spanish projects RTA2013-00091-C02-01/-02 and ERDF funds, and by the Community of Madrid (Spain) (project AGRISOST- S2018/BAA-4330) and Structural Funds 2014–2020 (ERDF and ESF).

### Data availability statement

Data included in article/supplementary material/referenced in article.

### Declaration of interests statement

The authors declare no conflict of interest.

### Additional information

No additional information is available for this paper.
